# Black soldier fly larvae protein production in Australia

**DOI:** 10.1093/af/vfad023

**Published:** 2023-08-14

**Authors:** K DiGiacomo

**Affiliations:** School of Agriculture and Food, Faculty of Science, The University of Melbourne, Parkville, Victoria 3010, Australia

**Keywords:** alternative protein, black soldier fly larvae (BSFL), feed, food, sustainability

**Implications**

- Insect production for human food and animal feed is increasing in Australia.
- Rearing insects from organic waste streams can contribute towards the sustainable development goals of the United Nations (UN).
- The Australian insect production industry remains small and in start-up stages, while increased collaborative and large-scale investment in research and development is occurring.
- The dominant insect grown in Australia is black soldier fly larvae, although no manufacturers are producing adequate volumes to meet market demands.

## Introduction

Insect production for food and animal feed in Australia has received increased attention and financial investment over the last decade. Australia is suited to insect production given the climate and strong history in agricultural production and research, with a valuable global reputation for producing safe food products. According to AgriFutures, the Australian insect protein industry has the potential to reach $10 million per annum within the next 5 years (Nolet, 2020), although to date the industry is emerging and remains relatively small, with limited research input compared to Europe and the United States. No doubt, the COVID-19 pandemic has driven delays in development due to disruptions to research and research output, changes to supply chains, and access to national and international products and experts. As such, the insect industry in Australia is entering a time where significant drive, investment, research, and outputs are required to advance this industry. Given the national and international interest in insect rearing and the renewed focus on sustainability, this is achievable, and it is likely that insect protein production in Australia will increase in the coming decade. In previous reviews we have covered the potential role of insect protein in Australian feed and food markets (DiGiacomo et al., 2019; DiGiacomo and Leury, 2019). This review will update (but not repeat) these previous reviews and examine the current state of insect protein research and production in Australia.

### Current insect production

**Figure 1.**
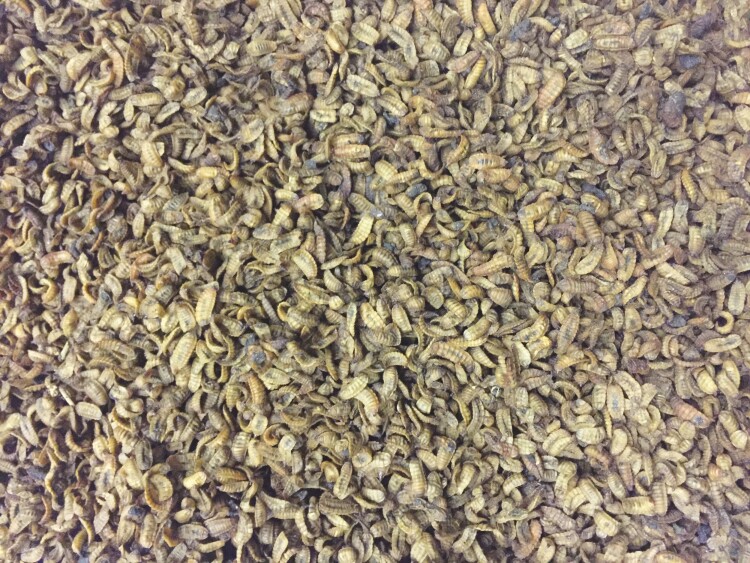
Oven dried black soldier fly larvae (BSFL) reared on food waste.

**Figure 2.**
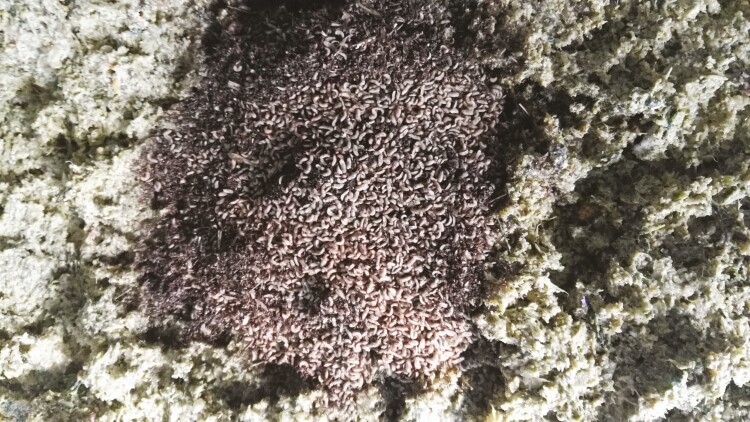
Processed food waste inoculated with black soldier fly larvae (BSFL) neonates.

The insect industry in Australia currently has 13 active companies (most of which are Australian owned; Table 1) and is dominated by producers of endemic insects such as the black soldier fly larvae (BSFL, Figures 1 and 2) for the animal feed industry, while most companies producing insects for human food are growing crickets (brown house cricket *Acheta domestica*). Membership of the Insect Protein Association of Australia (IPAA) has grown from 13 members to 57 in 2022 (22 commercial members, 9 primary producers, 23 individual members, and academics, students, consultants, and downstream businesses). Given the commercial nature of the industry, it is impossible to ascertain the current production volumes across Australia. However, large scale production is not yet underway. In a report produced by AgriFutures, only 35% of the insect production industry in Australia has 3–15 employees, with most having 1–3, and remaining in the start-up stages (Nolet, 2020). The lack of scale in the industry is driven by numerous factors including technology and engineering controls, access to capital investment, lack of customer base given the small-scale volumes presently produced, and lack of knowledge or research specific to Australian specific environments and systems.

**Table 1.** List of companies producing insects (black soldier fly larvae (BSFL), *Hermetia illucens*), crickets (brown house cricket *Acheta domestica*), mealworms (*Tenebrio molitor*), or ants (green tree ants *Oecophylla smaragdina*) for human food or animal feed purposes in Australia

Company	Year started	Location	Insect type	Notes
Animal feed				
Bardee	2019	Vic	BSFL	
Crickets & Co	2020	NSW	Crickets	
FlyFarm	*	Qld	BSFL	Hong-Kong company with Brisbane rearing facility
Future Green Solution	2012	WA	BSFL	
GoTerra	2015	ACT	BSFL	
Hatch Biosystems	2016	Vic	BSFL	
Karma 3	*	Vic	BSFL	No longer operating
Solution Blue	2020	NSW	BSFL	
SymbioPro	2015	Vic	BSFL	
Waste Not Food Recycling	2020	WA	BSFL	Not yet producing
Human food				
Something Wild	2016	SA	Native ants	
Grubs Up	2016	WA	Crickets, mealworms	
Circle Harvest (formerly Edible Bug Shop)	2007	NSW	Crickets, mealworms	
Schubugs/Grub Protein	2018	SA	Crickets	
Rebel Foods Tasmania	2018	Tas	Crickets, mealworms	No longer operating (2021)
Hoppa	2019	NSW	Crickets	

Information sourced from internet searches and media posts.

* = year unknown. ACT: Australian Capital Territory; NSW: New South Wales; SA: South Australia; Tas: Tasmania; Vic: Victoria; WA: Western Australia.

The Australian systems producing insects for animal feed are primarily focused on reducing waste and therefore utilizing food/feed waste as rearing substrates The Australian insect industry could contribute approx. 435,000 tonnes/year of insects to production animal feed (not including ruminants) (Nolet, 2020). Given the total volume of animal feed produced is approx. 13.6 million tonnes/year (Stock Feed Manufacturers’ Council of Australia, n.d.), insects would only contribute ~3% of this produced feed. At present large supermarket chains like Woolworths (Australia’s largest supermarket chain) are committing to reduce food waste destined to landfill by initiating waste diversion programs, redirecting surplus food to rescue organizations and waste/surplus food to farmers for use as animal feed or for processing into green energy or fertilizer (including insect rearing) by commercial contractors.

While the current focus of the Australian insect industry is producing insects for food/feed, high value products (e.g., chitin, frass, oils, antimicrobial peptides etc.) can all be sourced from insect production systems and these products may become a larger focus of the industry in the future (DiGiacomo et al., 2019; DiGiacomo and Leury, 2019).

There has been continued media interest in insect production, with a news story generally published each month covering some aspect of the industry (both journalistic pieces and those driven by commercial companies/marketing exercises). While this publicity is beneficial for industry members, it now needs to be closely backed by evidence of success and sustained production to garner continued investment and foster trust. According to a report by AgriFutures who conducted interviews (*n* = 30) with domestic and international insect producers, industry bodies and experts, consultants, and researchers (Nolet, 2020); the three key areas that should be the focus of the Australian insect production industry are 1) investment in industry convening initiatives; 2) the development of industry guidelines (which presently do not exist); and 3) investments into prioritized foundational research initiatives. Task groups including government (Animal Health Committee, Department of Agriculture) and industry (IPAA, Animal Health Australia etc.) representatives are examining the biosecurity risk of insects as animal feed and have submitted a white paper to the federal government with recommendations (no responses have been provided at the time of writing). Currently, insects have clear regulation when consumed as food (The Food Standards Australia & New Zealand Advisory Committee on Novel Foods), but their use as feed has unclear or nonexistent guidelines. For example, feeding insects to aquatic animals have clear permissible guidelines for all states and territories in Australia, while feed for poultry, swine, and ruminants are less clear. Currently, insects cannot be fed to ruminants as they come under the restricted animal material (RAM) guidelines. Swine can consume insects in Australia if the insects are not reared on any material containing swill or animal products (or only containing rendered/treated animal products). Insects can also be fed to poultry with some similar restrictions as noted in pigs for Queensland and South Australia. Insects remain excluded from the Australian animal welfare code, but changes to this may constrain research and industry development.

### Large scale research in Australia

The Fight Food Waste Cooperative Research Centre (CRC) with 59 participants (45 industry, 6 state and territory governments, and 8 universities) was established in 2018, receiving $120 million AUD funding over 10 years. The goals of the program are to develop and advance methods to reduce the $37 billion AUD in food waste produced from paddock to plate each year (Fight Food Waste CRC, 2021). The CRC estimates that by 2050 they will have contributed to the development of 344 jobs in the circular economy, a reduction in food waste of 11 million tonnes, an increased industry profitability to $1 billion AUD, and a greenhouse gas emission reduction of over 20 million CO_2_ equivalents (Fight Food Waste CRC, 2021). The CRC is examining the production of BSFL for livestock feed with a focus on identification of roadblocks for production, developing screening methods to discover and control waste streams by removing contaminants, identifying food waste volumes, locations, and potential risks for inclusion as pig feed.

Another large collaborative research program is underway (“Closing the loop, Black Soldier Fly technology to convert agricultural waste into high quality fertiliser and soil improvers from DAWR”; $2.5 million AUD, 2019–2023) between the University of Western Australia in collaboration with Australian Pork Limited (APL), Future Green Solutions (commercial partner), AgriFutures Australia, Australian Eggs, the Australian Meat Processor Corporation, Dairy Australia, the Department of Agriculture and Fisheries Queensland, and the Fisheries Research and Development Corporation. This project is examining a range of areas related to BSFL production including mapping waste locations and volumes, looking at attitudes, and willingness to use BSFL to manage waste, determining market potential, looking at engineering controls, and examining the use of frass as fertilizers and soil improvers. As indicated by the range of industries represented in this research, it is clear there is considerable interest in the production of insects for feed in Australian systems.

According to a recent roadmap produced by the Commonwealth Scientific and Industrial Research Organisation (CSIRO, Australia’s federal research organisation) the advancement of the Australian insect industry must 1) create new collaborations and partnerships with First Nations Peoples, industry, researchers, and government bodies; 2) codevelop First Nations owned and led initiatives, improve Western perceptions of insect consumption, and create Australian-branded food experiences; 3) identify and incorporate insect species already adapted to the Australian climate; and 4) produce new edible insect foods that are delicious, nutritious, and easy to access (Ponce-Reyes and Lessard, 2021). While Australia has >60 native insects recorded as being a traditional food for Indigenous Australian people (Ponce-Reyes and Lessard, 2021), to date there is no evidence of commercial farming of native/traditional Australian insects, and any investment in this area must be led by First Nations Peoples in a way that maintains cultural intellectual property and protects insect species with cultural significance. Thus, as well highlighted by the CSIRO roadmap, First Nations Peoples of Australia must be at the forefront of the development of the Australian insect industry and need to be involved in all stages of planning, development, and execution (Ponce-Reyes and Lessard, 2021). It remains unclear if such initiatives are currently underway but further investment in this area is crucial.

### Insects in domestic pet food

It is estimated that 61% of households in Australia have a pet, with approx. 5.1 million pet dogs, and 3.8 million pet cats in Australia (Animal Medicines Australia Pty Ltd., 2019). Yearly spending on food for pets in Australia is estimated at $3.9 billion AUD and is continuing to grow (Animal Medicines Australia Pty Ltd., 2019). Insects are produced for exotic pets (amphibians, snakes etc.) while the market for insects in dog and cat food products in Australia is small and growing. More suppliers are developing products and large retail groups such as the largest online pet supplies store “Pet Circle” are seeing increased interest in such products. At the time of writing this manuscript Pet Circle lists one insect dry dog food containing BSFL protein that is sold out, two dog treat products containing crickets (one sold out; no information on the species provided), and one company selling three dog treat products that list ‘insect protein’ on the ingredients list but give no additional information of insect type or inclusion rate. Insects can be included as an alternative protein to deal with allergies or to either partially or fully replace ‘traditional’ protein sources in the diet. At present, none of the larger pet food manufacturers in Australia are manufacturing or selling insect-based feeds. This is likely to change given that Mars Petcare in the United Kingdom are selling ‘Lovebug’, an insect-based cat food using insects produced by Protix based in the Netherlands. And similarly, Nestlé Purina is producing insect and plant protein-based pet food in Switzerland. Pet food regulations differ between each state and territory in Australia, and the Australian pet food industry is self-regulated by the Pet Food Industry Association Australia (PFIAA). Meat for pet consumption is covered by the Australian Standard (AS5812 for retail sale and good manufacturing practice compliance) though specific regulation on the inclusion of insects in pet feed is currently nonexistent.

As summarized by Kępińska-Pacelik and Biel (2022), while limited data exists published work suggests that incorporating insects as a portion of the diet for dogs does not negatively impact palatability, digestibility, or fecal consistency. In unpublished research from our group (Kerr et al., 2020) preference testing of dried BSFL (microwave dried, unprocessed, and whole) compared to commercial dog treats (beef, lamb, pork, chicken, fish, or sweet potato products) indicated that dogs preferred the traditional protein sourced treats, although dogs were interested in the BSFL and some dogs preferentially selected insects (particularly when selecting between sweet potato or BSFL). This was a small masters research experiment and did not attempt to quantify the previous food experiences of the dogs tested and was further limited by the comparison of processed traditional proteins compared to whole unprocessed BSFL. Consumer acceptance of insects in pet feed is a larger barrier but many owners do show interest in feeds containing insects and it is likely that exposure and education can increase consumer demand for such products (DiGiacomo et al., 2019; Kępińska-Pacelik and Biel, 2022).

### Edible insects for human consumption

There are a limited number of insect products currently produced commercially for human consumption in Australia (Table 1), and they mainly include flours, dried insects, and protein meals. Globally there are significantly more insect-based products on the market such as beers and other drinks, burgers, and alternative meats, pastas and breads, cookies, chocolates, ice creams, butters, and oils. It is likely that some of these locally produced products could be successfully produced and sold to Australian consumers in the coming years.

In a recent online survey of Australian consumers, 35% had previously consumed insects while 56% were willing to try insects in the future (Hopkins et al., 2022). After reviewing 70 published articles Testa et al. (2017) found that the greatest risks or perceived risks for the safety of insects as human food or animal feed related to allergy, microbiological and chemical contamination, malabsorption, hematic alteration, and the role of antinutrients. For Australian consumers the largest barriers to consumption were identified as lack of opportunity (57%), lack of trust (16%), and those who don’t eat animals (8%); while participants indicated that the greatest motivating factors would be knowledge, accessibility, safety endorsement, and regular appearance in mainstream media (Hopkins et al., 2022). While this information provides valuable insights into the mindset of Australian consumers, the findings are not unexpected, and the insect production industry is aware of such challenges. Nevertheless, the lack of consistent production currently precludes industry wide investment in marketing and education type programs, and any such activities are presently grass roots level and small scale.

Food safety, including preparation and processing methods, is of utmost importance and producers need to ensure insect products meet regulatory and consumer demands. Recent Australian research found that BSFL reared on food waste (preconsumer meat-free waste) had high, but acceptable (from a regulatory basis) total viable counts after freezing, perhaps suggesting that the frozen product would not have a long shelf life (Bessa et al., 2021). The presence of endospores was below regulatory limits, and while *Salmonella* and *L. monocytogenes* were not detected, *E. coli* was found at above acceptable levels (for minced meat) in frozen but not blanched samples (Bessa et al., 2021). This study concluded that rearing substrate did not influence microbial loads of the BSFL, while killing method (blanching vs. freezing) had more of an impact, which was also true for heavy metal and mineral concentrations (Bessa et al., 2021). It is unlikely that raw insect products would be sold for human consumption and thus drying and processing treatments can be utilized to ensure product safety. Further, as insects produced for human consumption are unlikely to be reared on nonfood grade or contaminated waste streams the likelihood of contamination is low. However, insects can accumulate heavy metals if they are present in the rearing substrate (Charlton et al., 2015). There is potential for insects to cause cross reactivity with allergens when consumed by humans, as noted by Bessa et al. (2021) who showed cross reactivity with crustacean allergens even though the BSFL were not fed crustaceans. This strongly suggests that consumers allergic to crustaceans may be allergic to BSFL, although more specific research in this space is warranted.

While it is unlikely that insect protein will overtake traditional animal-based protein sources (i.e., meat and milk products) for food (and feed), diversification of food chains is required to reach sustainability goals. Further, variety in choices for feed and food substrates are important so producers and consumers can make decisions about what they produce and eat. Consumers are increasingly interested in sustainability and understanding where their food comes from. The sustainable production of animal derived products may therefore become a key marketing position for promoting domestic and international sales in the future and feeding insects as animal feed or rearing insects on waste may contribute to this goal.

### Competition for waste

There are numerous methods via which organic waste can be utilized in Australia, including use as animal feed, donations to food rescue services, processing as waste to energy via anaerobic codigestion, processing for use in other foods, commercial or at home composting, processing into nonfood products, or directed to on farm or commercial landfill. Risks for insect production therefore include challenges around consistent rearing substrate supply. Organic waste and by-product production is impacted by climatic, social, economic, and trade activities. This instability of substrate production may contribute towards instability of insect production in Australia. For large producers to commit to using insects in their diets insect production would need to increase and be consistent, and it is unlikely that insect protein production volumes could totally replace the use of traditional proteins in animal diets. However, any contribution that can improve the sustainability and environmental impact will have significant positive outcomes for the industry.

Estimates on actual usage of food waste as animal feed are difficult to accurately ascertain, with the national food waste baseline recently estimating that of the >7.5 million tonnes of waste arising across the food chain in Australia, approx. 70% of this waste is considered edible and a significant portion is already redirected to animal feed (~6.8 million tonnes) (Bontinck and Grant, 2021). Of this waste, approx. 1 million tonnes of the 1.75 million tonnes of waste produced from the prefarm gate production is lost in on farm disposal (Bontinck and Grant, 2021). The greatest total losses were estimated to occur from consumer household and hospitality waste, as well as at manufacturing (Bontinck and Grant, 2021). However, accessing household and restaurant waste streams is likely to be logistically and financially difficult. The most suited targets for waste streams to supply insect production are thus likely to come from prefarm gate by redirecting on-farm disposal towards insect production.

As part of a larger research project farm animal waste volumes in Australia were estimated (from 2010 to 2019) across the dairy, pig, poultry, and meat processing sectors; finding that a combined total of ~2.6 million dry tonnes of farm waste is produced per year (Kragt et al., 2021a). This waste varies state by state based on locations and density of primary producers. For example, Victoria produces the largest volume of dairy waste while Queensland produces the most piggery, poultry, and meat processing waste (Kragt et al., 2021a). Thus, there will be regional differences in access to waste (including primary product, processing waste, and manure). This will impact the processing of insects using food waste as a substrate and will further influence the nutritional profile of the resultant insects as it is well established that rearing substrate influences nutritional profiles of insects (Jucker et al., 2017; DiGiacomo et al., 2019; Oonincx and Finke, 2021). Further, some waste products are either not suitable for use without processing or require a combination of ingredients to be suitable (i.e., overly wet products like liquid milks) for use as insect feed. For example, in the red meat sector, most of the waste is generated at the abattoir, though much of the waste produced is high in fat and nitrogen. We recently reported that low-value pork processing waste (trim, offal, testes, skin, throat, and uncleaned large intestine) while able to somewhat be consumed by BSFL was unable to adequately sustain larvae growth without the addition of a carbohydrate source (DiGiacomo et al., 2021). However, blending combinations of waste to achieve uniformity in nutrient profile and substrate consistency would require additional resources (labor and equipment) that would likely increase production costs for insect manufacturers.

As discussed in previous reviews (DiGiacomo et al., 2019; DiGiacomo and Leury, 2019) manure is a potential nutrient source for insects in Australia. A recent study attempted to quantify the cost of management of manure produced by the Australian pig production industry, finding that highly variable management practices make quantification difficult, and presently little information exists (Kragt et al., 2021b). This research identified both financial and nonmarket costs associated with manure management which included freight costs, labor, environmental costs (groundwater contamination, GHG emissions, odor generation etc.) (Kragt et al., 2021b), which would all need to be considered before insect rearing can occur.

**Figure 3.**
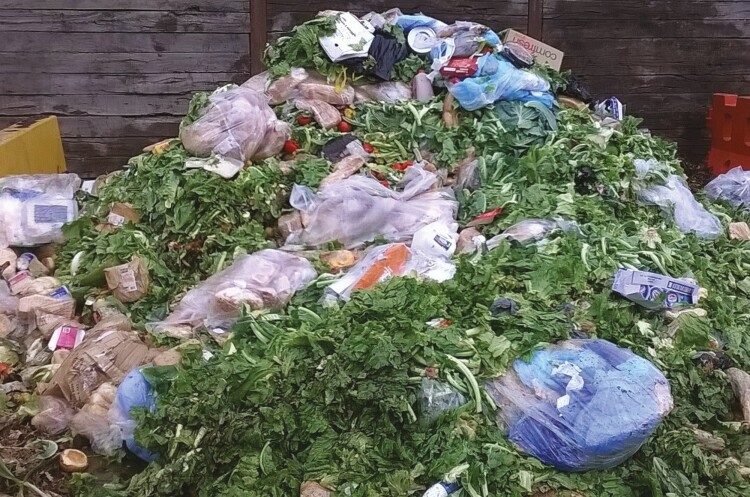
Waste received from a local supermarket for processing as a substrate for black soldier fly larvae BSFL rearing.

Processing of waste also needs to be considered. As evident in Figure 3, which is an example of supermarket waste provided to an insect production facility in Australia, mixed waste from supermarkets may require significant processing and handling prior to feeding to insects. The waste shown in Figure 1 is a mix of vegetable and bakery waste, but also contains packaging like cardboard and plastic. Recent research demonstrated that BSFL growth rate and substrate reduction rate, but not survival, was reduced when plastic (microplastics of polyethylene and polystyrene at 5, 10, or 20% inclusion rate) was included in the rearing substrate (Cho et al., 2020). In the same study BSFL growth was more significantly restricted when fed high salt diets compared to those containing plastics (Cho et al., 2020). Australian research found that feeding plastic (various types) didn’t significantly alter BSFL or mealworm growth rates (except for one type of plastic porous polylactic acid blocks that reduced BSFL weight by ~29%) or survival (Beale et al., 2022). While there is potential for small volumes of plastics to be acceptable for inclusion in BSFL diets, these studies did not examine the safety of the resultant larvae so additional research would be required to ensure contamination of feed/food is not occurring.

### Workforce constraints?

There has been a consistent decline in the agricultural workforce in OECD countries which has led to farmers being unable to meet their labor needs. This is driven by complex factors that cannot be covered in this review but includes issues such as a drive for higher paying and less laborious jobs, migration away from rural areas, and responses to the industrialization of farming (Dedieu et al., 2022). One of the identified key cross-cutting issues in agricultural workforces is the need to bring agricultural work closer to the UN sustainable development goals (Lee et al., 2016) which includes “decent employment” for all workers. The insect production industry is in a rare space given its infancy and given that the industry was essentially exclusively developed to achieve sustainability goals. While the insect production industry is exploring the development and use of automated or robotic production systems, such automation will likely shift labor requirements to a different role/focus, rather than eliminating the requirement for workers. This unique space gives the industry the potential to contribute to numerous sustainability goals including those related to workforce development and worker satisfaction as well as those more directly related to food/feed production and environmental sustainability. There is an opportunity for this industry to become one of the most diverse and inclusive in Australia. This may include diversity in gender (anecdotally the Australian insect production industry is dominated by females) and diversity in cultural background such as the inclusion of First Nations peoples. There is potential for the industry to embrace a diversity of locations (i.e., not necessarily concentrated in regional areas), attract a wide range of skills (e.g., digital/precision agriculture), and attract staff interested in contributing towards a more sustainable agricultural industry. This high level of diversification may contribute to increases worker satisfaction and retention, although specific research on insect industry worker satisfaction is nonexistent.

### Other challenges

Many of the challenges faced by the Australian insect production industry are not unique, such as those associated with engineering controls and scaling up, while others are more specific to the Australian system. One of the larger constraints is government regulation and inconsistent rules between states and territories which limits production scaling. The ambiguity also creates risk for some markets, with human food products, poultry, and aquaculture presently the easiest target industries in terms of regulation. The increase in interest for use in pet foods is a likely useful market but given the pet food industry in Australia is self-regulated there are risks associated with this lack of regulation if appropriate controls are not in place. Numerous constraints for the use of Insects in human food remain and include potential contamination and allergens, consistent production, potential religious, and philosophical constraints, and other public concerns such as worries about rearing and where facilities are located.

The industry is currently constrained by a lack of collaboration due to real and/or perceived risk of nonmutual benefits (i.e., one party will have a competitive advantage over the other) (Nolet, 2020; Ponce-Reyes and Lessard, 2021). In addition, insect research facilities are minimal for noncommercial enterprises. Research at an industry level is well coordinated in some ways, as evidenced by some larger scale funded research described previously, yet remains in its infancy. Continued investment in collaborative research projects (across insect producers, states, universities, agricultural and government industries) is imperative. The insect industry in Australia is also constrained by competition both domestically and internationally. Australia has is expensive in terms of infrastructure, energy costs, land value, and human labor costs. Thus, compared to other countries the insect production industry in Australia may be less competitive. For example, it might be less cost effective to divert waste streams towards insect production unless incentives such as tax breaks are introduced.

## Conclusion

The Australian insect production industry remains in the start-up stages but has a unique opportunity to develop as a world leading diverse, sustainable, inclusive, and profitable industry that assists in dealing with challenges from other agricultural sectors including waste management and resource availability. The current challenges faced by the Australian insect industry can be addressed by further investment in infrastructure, people, education, marketing, and research. The growth of the industry needs to be well supported by government regulations to ensure safety and inconsistency in these regulations is currently limiting efforts in collaboration and production. Recent large-scale investments into collaborative research programs are an excellent starting point from which the industry can grow, while further investments are needed.

*Conflict of interest statement*. None declared.

## About the Author

**Dr Kristy DiGiacomo** is a senior lecturer in production animal nutrition at the University of Melbourne, Australia. She earned both her Bachelor of Animal Science and Management (Honours) and PhD (The physiological and metabolic responses to heat in ruminants) from the University of Melbourne while completing part of her PhD with the University of Arizona. She has since been employed as a postdoc then lecturer in animal nutrition and physiology at The University of Melbourne. Kristy is a Veski sustainable agriculture fellow. Her research areas include livestock nutrition, alternative protein feeds (inc. insects), dairy animal efficiency, animal physiology, and animal adaptation to the external environment.
